# Supplementary material for: Urban Stormwater Resilience Assessment Method Based on Cloud Model and TOPSIS
Source: Int J Environ Res Public Health. 2021 Dec 21;19(1):38. doi: 10.3390/ijerph19010038 (PMC8751000; doi:10.3390/ijerph19010038)
Supplement: Supplementary file 1 [file ijerph-19-00038-s001.zip › ijerph-1484791-supplementary.pdf]

The C_2 value standard of flood control embankment length: whether the total length of urban flood control embankment conforms to the design code of urban flood control embankment.

Table S1. Fuzzy description of the length of the levee

Length of the levee C_2	Scoring criteria	Score
Level 1: The worst plan Y^-	Length<10km	1
Level 2	Length between 10km and 15km	2
Level 3	Length between 16km and 12km	3
Level 4: The best plan Y^+	Length>20km	4

Criteria for the C_3 value of drainage pipe length: whether the total length of drainage pipe conforms to the urban drainage engineering planning standards.

Table S2. Fuzzy description of drainage pipe length

Length of the drainage pipe C_3	Scoring criteria	Score
Level 1: The worst plan Y^-	Length<5000km	1
Level 2	Length between 5000km and 10000km	2
Level 3	Length between 10000km and 15000km	3
Level 4: The best plan Y^+	Length>15000km	4

The value standard of pumping and discharging capacity C_4 of central urban water pump: the total pumping and discharging capacity of central urban water pump station.

Table S3. Fuzzy description of pumping and discharging capacity of central urban water pump

Pumping and discharging capacity of central urban water pump C_4	Scoring criteria	Score
Level 1: The worst plan Y^-	Capacity<$400m^3 \cdot s^{-1}$	1
Level 2	Capacity between $400m^3 \cdot s^{-1}$ and $600m^3 \cdot s^{-1}$	2
Level 3	Capacity between $600m^3 \cdot s^{-1}$ and	3

	$800m^3 \cdot s^{-1}$	
Level 4: The best plan Y^+	Capacity>$800m^3 \cdot s^{-1}$	4

Value standard of road area per capita C_5: road area per capita calculated according to urban population. The overall well-off target value is $12m^2$.

Table S4. Fuzzy description of per capita road area

Per capita road area C_5	Scoring criteria	Score
Level 1: The worst plan Y^-	Per capita road area<$4m^2$	1
Level 2	Per capita road area between $4m^2$ and $8m^2$	2
Level 3	Per capita road area between $8m^2$ and $12m^2$	3
Level 4: The best plan Y^+	Per capita road area>$12m^2$	4

Average number of students in institutions of higher learning per 10,000 population C_6 value standard: number of students in institutions of higher learning per 10,000 population.

Table S5. Fuzzy description of the average number of students in institutions of higher learning per 10,000 population

Average number of students in institutions of higher learning per 10,000 population C_6	Scoring criteria	Score
Level 1: The worst plan Y^-	The number of students<1000	1
Level 2	The number of students between 1000 and 1500	2
Level 3	The number of students between 1500 and 2000	3
Level 4: The best plan Y^+	The number of students>2000	4

Employment rate C_8 value standard: the proportion of urban employment in the total number of people.

Table S6. Fuzzy description of employment rate

Employment rate C_8	Scoring criteria	Score
Level 1: The worst plan Y^-	Employment rate<70%	1
Level 2	Employment rate between 70% and 80%	2
Level 3	Employment rate between 80% and 90%	3
Level 4: The best plan Y^+	Employment rate>90%	4

The value standard of urban medical insurance popularity C_9: the proportion of the number of people covered by urban medical insurance to the total urban population.

Table S7. Fuzzy description of urban medical insurance popularity

Urban medical insurance popularity C_9	Scoring criteria	Score
Level 1: The worst plan Y^-	Popularity<70%	1
Level 2	Popularity between 70% and 80%	2
Level 3	Popularity between 80% and 90%	3
Level 4: The best plan Y^+	Popularity >90%	4

Value standard of C_{10} for health beds per 1000 population: the number of health beds per 1000 population in medical and health institutions.

Table S8. Fuzzy description of sanitary beds per 1000 population

Sanitary beds per 1000 population C_{10}	Scoring criteria	Score
Level 1: The worst plan Y^-	Sanitary bed number<4	1
Level 2	Sanitary bed number between 4 and 5	2
Level 3	Sanitary bed number between 5 and 6	3
Level 4: The best plan Y^+	Sanitary bed number>6	4

Number of health workers per 1000 population C_{11} value standard: number of health workers per 1000 population.

Table S9. Fuzzy description of the number of health workers per 1,000 population

Number of health workers per 1,000 population C_{11}	Scoring criteria	Score
Level 1: The worst plan Y^-	Number of staff<10	1
Level 2	Number of staff between 10 and 15	2
Level 3	Number of staff between 15 and 20	3
Level 4: The best plan Y^+	Number of staff>20	4

Value standard of green coverage rate C_{13} of built-up area: the percentage of green coverage area of urban built-up area in built-up area.

Table S10. Fuzzy description of greening coverage rate in built-up areas

Greening coverage rate in built-up areas C_{13}	Scoring criteria	Score
Level 1: The worst plan Y^-	Greening coverage rate in built-up areas<20%	1
Level 2	Greening coverage rate in built-up areas between 20% and 30%	2
Level 3	Greening coverage rate in built-up areas between 30% and 40%	3
Level 4: The best plan Y^+	Greening coverage rate in built-up areas>40%	4

Lake-river surface area C_{14} value standard: the total surface area of rivers and lakes.

Table S11. Fuzzy description of lake and river surface area

Lake and river surface area C_{14}	Scoring criteria	Score
Level 1: The worst plan Y^-	Lake and river surface area<10km^2	1
Level 2	Lake and river surface area between 10km^2 and 30km^2	2
Level 3	Lake and river surface area between 30km^2 and 50km^2	3
Level 4: The best plan Y^+	Lake and river surface area>50km^2	4

Paddy area C_{15} value standard: the size of the land area used for paddy field cultivation in the city.

Table S12. Fuzzy description of paddy farmland area

Paddy farmland area C_{15}	Scoring criteria	Score
Level 1: The worst plan Y^-	Paddy farmland area<1000km^2	1
Level 2	Paddy farmland area between 1000km^2 and 2000km^2	2
Level 3	Paddy farmland area between 2000km^2 and 3000km^2	3
Level 4: The best plan Y^+	Paddy farmland area >3000km^2	4

Reservoir capacity C_{16} value standard: urban reservoir total capacity value.

Table S13. Fuzzy description of reservoir capacity

Reservoir capacity C_{16}	Scoring criteria	Score
Level 1: The worst plan Y^-	Reservoir capacity<100 billion m^3	1
Level 2	Reservoir capacity between 100 billion and 250 billion m^3	2
Level 3	Reservoir capacity between 250 billion m^3 and 500 billion m^3	3
Level 4: The best plan Y^+	Reservoir capacity>500 billion m^3	4

The value standard of C_{17} for comprehensive production capacity of water plant: the comprehensive production capacity of urban water plant.

Table S14. Fuzzy description of comprehensive production capacity of water plant

Comprehensive production capacity of water plant C_{17}	Scoring criteria	Score
Level 1: The worst plan Y^-	Comprehensive production capacity<10 million t·d^{-1}	1
Level 2	Comprehensive production capacity between 10 million t·d^{-1} and 50	2

	million $t \cdot d^{-1}$	
Level 3	Comprehensive production capacity between 50 million $t \cdot d^{-1}$ and 100 million $t \cdot d^{-1}$	3
Level 4: The best plan Y^+	Comprehensive production capacity>100 million $t \cdot d^{-1}$	4

Wastewater treatment capacity C_{18} value standard: sewage treatment plant daily capacity of sewage.

Table S15. Fuzzy description of sewage treatment capacity

Sewage treatment capacity C_{18}	Scoring criteria	Score
Level 1: The worst plan Y^-	Sewage treatment capacity<2 million $m^3 \cdot d^{-1}$	1
Level 2	Sewage treatment capacity between 2 million $m^3 \cdot d^{-1}$ and 3 million $m^3 \cdot d^{-1}$	2
Level 3	Sewage treatment capacity between 3 million $m^3 \cdot d^{-1}$ and 4 million $m^3 \cdot d^{-1}$	3
Level 4: The best plan Y^+	Sewage treatment capacity>4 million $m^3 \cdot d^{-1}$	4
